# Gastric and adrenal metastasis from breast cancer: Case report and review of literature

**DOI:** 10.1097/MD.0000000000018812

**Published:** 2020-01-17

**Authors:** Tiantian Tang, Lina Zhang, Chunxiao Li, Tao Zhou

**Affiliations:** Breast Center, The Fourth Affiliated Hospital of Hebei Medical University, Shijiazhuang City, Hebei Province, PR China.

**Keywords:** adrenal metastasis, breast cancer, diagnosis, gastric metastasis, GATA3, mammaglobin

## Abstract

**Rationale:**

Breast cancer is the most commonly diagnosed malignancies in females. The most common sites of metastasis are bone, lung, liver, and brain. Gastrointestinal and adrenal gland metastasis from breast cancer are rare. Simultaneous metastases are extremely rare. Therefore, it is critically important to choose proper examination and treatment since the rapid diagnosis and primary treatment can significantly affect the survival of patients. To the best of our knowledge, this was the first case of initial dual metastasis.

**Patient concerns:**

The patient had a history of left breast cancer, and she underwent left breast-conserving surgery with sentinel lymph node biopsy 2 years ago. She was hospitalized in our center with the complaints of a stomach and lower back pain, which started suddenly and was progressively increased for half a month.

**Diagnosis:**

Computed tomography, gastroscopy, and immunohistochemical staining, especially GATA3 and mammaglobin, confirmed that there was simultaneous gastric and adrenal metastases.

**Interventions:**

She was eligible for the IMpassion131 clinical trials, a Phase 3 randomized, double-blind, placebo-controlled trial under treatment with atezolizumab/palcebo plus paclitaxel as adjuvant-therapy.

**Outcomes:**

She was still undergoing the therapy and waiting for the further evaluation.

**Lessons:**

In order to better understand metastatic pathways of breast carcinoma, publications of individual patient cases diagnosed with rare metastatic sites should be encouraged, especially for the simultaneous rare metastatic sites. This might improve our understanding of metastatic behavior of breast cancer and promote further clinical research.

## 1 Introduction

Breast cancer is the most frequently diagnosed cancer among women. Although its 5-year survival rate is increasing in recent years due to surgery, chemotherapy, endocrinotherapy, targeted molecular therapy, and radiotherapy, relapses still have been reported in 30% to 80% of patients with breast cancer.^[1]^ The most common sites of distant metastasis include lung, bone, liver, and brain. The incidence of gastrointestinal (GI) tract metastasis varies from 4% to 18%,^[2–5]^ and adrenal metastasis is also rare.^[6–9]^ Nevertheless, coexisting solitary metastases to both stomach and adrenal glands are extremely rare. In the present study, we reported a very rare case of metastasis in both stomach and adrenal gland from breast cancer and summarized the relevant literatures. To the best of our knowledge, this was the first case of a synchronous spread to stomach and adrenal glands as the first and unique initial manifestation of metastatic breast cancer.

## 2 Case report

**Figure 1:**
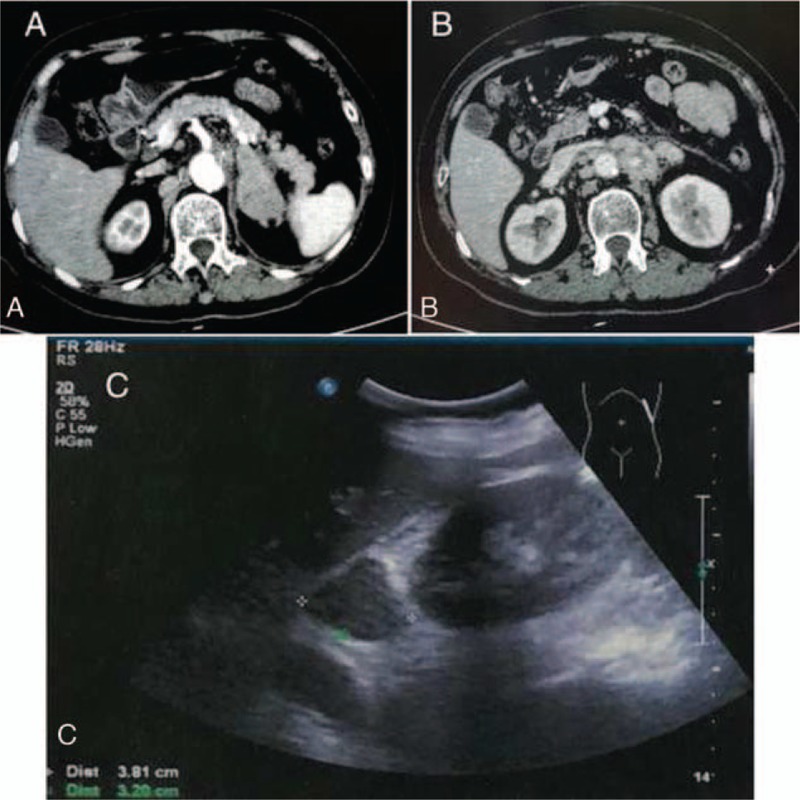
(A) Enhanced CT scan showing a 5.1 × 3.0 cm, uneven enhanced, rounded mass in the left adrenal gland. (B) Enhanced CT scan showing enlarged lymph nodes in the abdominal cavity and behind peritoneum. (C) Ultrasonography showed a 3.8 × 3.4 × 3.2 cm mass on the left adrenal gland.

A 67-year-old Chinese female was hospitalized in our center with the complaints of a stomach and lower back pain, which started suddenly and was progressively increased for half a month in Mar. 2019. The patient had a history of left breast cancer, and she underwent left breast-conserving surgery with sentinel lymph node biopsy (SLNB) in October 2017. Postoperative histopathological examination revealed an original invasive ductal carcinoma (IDC) of grade III (Fig. 1) with a size of 2.2 × 1.8 × 1.8 cm. The sentinel lymph node was 3/5 positive (the maximum diameter was 3–15 mm), axillary lymph node dissection (ALND) was performed, and the remaining axillary lymph node was 0/9 positive. Immunohistochemical (IHC) evaluation showed that estrogen receptor (ER), progesterone receptors (PR), and human epidermal receptor (HER-2) were negative. Therefore, it was considered as stage IIb with T2N1M0. Adjuvant chemotherapy based on 50 mg/m^2^ pirarubicin and 600 mg/m^2^ cyclophosphamide every 21 days for 4 cycles was finished in April 2018, followed by 4 courses of 100 mg/m^2^ docetaxel every 21 days. Radiotherapy was administered to the remaining breast and drainage areas. The patient was followed up every 3 months, and there were no relapses until March 2019.

**Figure 2:**
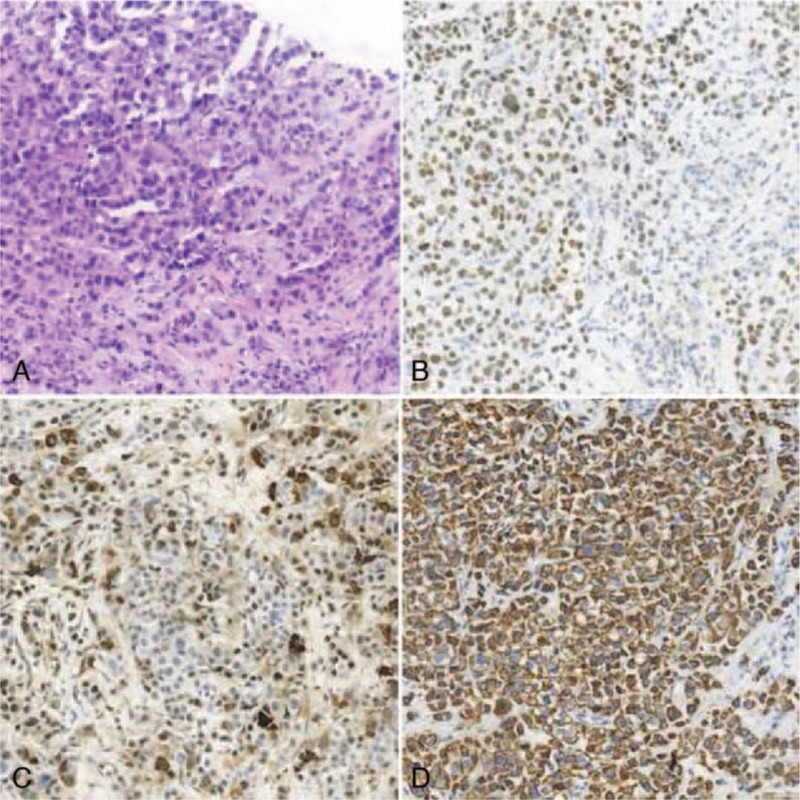
(A) Original magnification × 200: HE staining showed low differentiated adenocarcinoma in the adrenal metastatic lesion. (B) Original magnification ×200: The tumor cells show positivity for GATA3 by IHC analysis in the adrenal metastatic disease. (C) Original magnification ×200: The tumor cells show positivity for mammaglobin by IHC analysis in the adrenal metastatic disease. (D) Original magnification ×200: The tumor cells show positivity for CK by IHC analysis in the adrenal metastatic disease.

**Figure 3:**
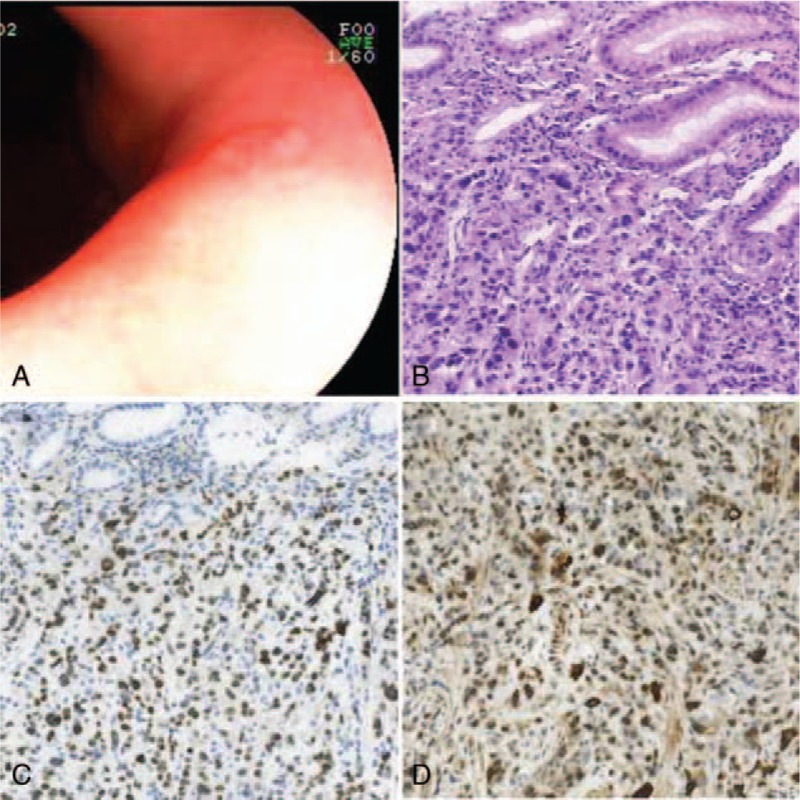
(A) Gastroscopy demonstrated a 0.8 cm in diameter, prominent nodule on the surface of the lesser curvature near the angle of the stomach. (B) Original magnification ×200: HE staining showed low differentiated adenocarcinoma in the gastric metastasis lesion. (C) Original magnification ×200: The tumor cells show positivity for GATA3 by IHC analysis in the adrenal metastatic disease. (D) Original magnification ×200: The tumor cells show positivity for mammaglobin by IHC analysis in the adrenal metastatic disease.

In March 2019, the patient revealed a stomach and lower back pain. Thoracic and abdominal enhanced computed tomography (CT) showed an uneven enhanced, rounded mass in the left adrenal gland, measuring 5.1 × 3.0 cm, which was considered as metastasis (Fig. 1A), with enlarged lymph nodes in the abdominal cavity and behind peritoneum (Fig. 1B). The left renal vein and inferior vena cava were normal. Ultrasonography showed a mass of 3.8 × 3.4 × 3.2 cm on the left adrenal gland (Fig. 1C). CT-guided biopsy of left renal mass was performed, and histopathological (HE) examinations confirmed a metastatic event from IDC of the breast since same characteristics of the tumor cells were observed (Fig. 2A). IHC staining on metastasized adrenal tumor showed negative for ER, PR, and HER2. Ki-67 was positive for 50%. Caudal type homeobox 2 (CDX2), thyroid transcription factor-1 (TTF-1), and melanoma antigen recognized by T-cells 1 (MART-1) were also negative, while GATA3 (Fig. 2B) and mammaglobin (Fig. 2C) were positive. CK was also positive (Fig. 2D), indicating the metastasis of breast cancer. Because of stomach ache, gastroscopy was performed, and results demonstrated a prominent nodule of 0.8 cm in diameter on the surface of the lesser curvature near the angle of the stomach (Fig. 3A), and the nodule was excised for pathological examination. Pathology report showed poorly differentiated adenocarcinoma (Fig. 3B). IHC staining was also negative for ER, PR, and HER2. Ki-67 was positive for 50%. CDX2, TTF-1, CEA, and Syn were also negative. GATA3 (Fig. 3C), mammaglobin (Fig. 3D), and CK were also positive. These findings further confirmed that the gastric metastasis also came from mammary gland.

Further examination revealed that the cardiac function of patient was normal. The blood test was carried out for tumor mark CA125, which was slightly above the normal range (44.11 U/ml; normal range: 0.0–35.0 U/ml). Moreover, the levels of CA15–3 (8.66 U/ml; normal range: 0.0–28.0 U/ml) and CEA (3.35 U/ml; normal range: 0.0–5 U/ml) were still within the normal range. Extensive imaging evaluations, including digital mammography, breast ultrasonography, head CT scan, and isotope bone scanning, revealed no other metastasis. She was eligible for the IMpassion131 clinical trials, a Phase 3 randomized, double-blind, placebo-controlled trial under treatment with atezolizumab/palcebo plus paclitaxel as adjuvant-therapy. The patient had no family history or other treatment history.

This study was proved by the Ethical Committee of our hospital, and patient provided informed consent for publication of the case.

## 3 Discussion

Single gastric^[10]^ or adrenal gland^[11]^ metastasis from breast cancer has been previously described. However, to the best of our knowledge, this was the first case of metastases in both locations and a synchronous spread to stomach and adrenal glands as the first and unique initial manifestation of metastatic breast cancer.

The incidence of gastric metastasis from breast cancer ranges from 2% to 18% in autopsy cases.^[12,2]^ However, Borst and Ingold have reported an extremely low rate of (0.26%, only 17 of 2604 cases) metastasis to the GI tract from breast cancer.^[8]^ McLemore et al have also shown a low rate (0.34%) of GI metastasis from breast cancer.^[13]^

The most common histological type of gastric metastasis from breast cancer is lobular carcinoma.^[5]^ However, some cases can also metastasize from ductal carcinoma.^[4]^ Nevertheless, in our case, the pathological subtype of the breast cancer was IDC of grade III.

The common sites of gastric metastasis include fundus and antrum, while such metastasis is unlikely to occur in both fundus and antrum simultaneously.^[4]^ It is generally presented with diffuse involvement of gastric wall, which is expressed as linitis plastica and mostly localized to the submucosal and seromuscular layers.^[12]^ The metastatic tumor mass of some patients is located at 1 focal area, and metastasis is also perforated from that focus.^[5]^ In our present case, there was only a single and very small nodule on the surface of antrum.

Symptoms from GI metastasis are various. Most patients usually suffer from non-specific GI symptoms, such as anorexia, upper abdominal pain, indigestion, nausea, pyrosis, and weight loss.^[12]^ Güler et al have reported that the patient with GI metastasis from breast cancer is admitted with an acute abdominal pain caused by the gastric perforation.^[5]^ Our patient also suffered from stomach ache, but it was not so severe.

Unlike the gastric metastasis, the rate of adrenal metastasis from breast cancer is even lower. Li et al have reported that the incidence of adrenal metastasis from breast cancer is 0.25% (34/13,595) in Chinese patients.^[14]^ Though the incidence of adrenal metastasis from breast cancer is lower, the adrenal gland is the first metastatic site in the uncommon metastatic sites from breast cancer.^[15]^

Symptoms of adrenal gland metastasis are always asymptomatic. If a large part of adrenal cortex is damaged by a tumor or both adrenal glands are affected, they can be associated with signs and symptoms of adrenal insufficiency.^[16]^ Our patient was also bothered by the lower back ache, and the pain was getting worse during the hospitalization. CT confirmed that the nerve was compressed because of the big metastatic tumor.

Most metastatic tumors from breast cancer are diagnosed by routine examination. Wrong diagnosis may delay the prior treatment of the disease, leading to unwanted morbidity and mortality. Therefore, it is critically important to choose a proper examination method. Usually, commonly used test methods are ultrasound and CT. If there are suspicious lesions, we should find a better examination mean. The sensitivity of PET has been reported to be relatively low for the diagnosis of gastric cancer compared with the other types of cancer. However, PET/CT scan has been reported to successfully identify adrenal metastasis.^[9]^ If there is any suspicious state of gastric diseases, esophagogastroscopy should be performed, which can find tiny lesions. However, although the imaging techniques are helpful in differentiating metastasis from primary tumor of metastatic tumor, the specificity of these imagine-based detections has been always an issue. The final diagnosis should depend on biopsy or metastasectomy. IHC staining of mammaglobin, GATA3 and gross cystic disease fluid protein-15 (GCDFP-15) is used to help diagnose metastatic tumor from breast cancer. Those are breast-specific antigens, which are accepted markers for epithelia of breast origin.^[17,18]^ GATA3 is reported to show a high sensitivity for nodal metastases and distant metastases, which is superior to GCDFP-15 and mammaglobin in identification of primary and metastatic breast cancer.^[19]^ In our patient, GATA3 and mammaglobin were positive both in metastatic lesions of gastric and adrenal gland, while the ER, PR, and HER2 staining were negative. Such findings were consistent with the IHC staining of primary breast cancer.

The purpose of recurrent and metastatic breast cancer treatment is to improve the quality of patient’ life and prolong the life of patients. Currently, there are no guidelines for the treatment of gastric metastasis or adrenal metastasis from breast cancer. There are even no guidelines for patients with simultaneous gastric and adrenal metastases from breast cancer. Systemic medical treatment is the main therapeutic regimen in most metastatic cases, such treatment should be given based on the hormonal receptor and HER2 status of primary and metastatic lesions, and general medical condition of the individual is also a key factor when making the therapeutic decision. McLemore et al have reported that surgical resection for the stomach actually does not have a significant impact on survival.^[13]^ However, surgical resection may play a role in palliative care for relief of symptoms, supportive care of the patients, or emergency situations where acute abdominal pain occurs.^[4,5]^ A single centre retrospective study by Maher et al has shown that among the 35 cases of gastric metastasis from breast cancer, 4 patients receive total gastrectomy.^[4]^ However, it is very regrettable that they have not reported why these 4 patients receive total gastrectomy, and we could not know the outcomes of these patients. Similarly, systemic therapy is also the first option for the adrenal metastasis from breast cancer. Several studies have reported that adrenalectomy for solitary adrenal metastasis results in a better survival in some patients, while such beneficial effect is only found in adrenal metastasis from colorectal cancer, lung cancer and gastric cancer^[20–22]^. Yoshitomi et al have reported a patient with adrenal metastasis from breast cancer, who receives adrenalectomy, followed by toremifene treatment as endocrine therapy, and this patient has 28 months of progression-free survival after the operation.^[23]^ Recently, some authors^[4,5,12]^ have advocated laparoscopic adrenalectomy (LA) for patients with solitary adrenal metastasis, while LA is limited by the tumor size. Usually, surgical excision is recommended in the situations as follows. The metastatic lesion is solitary and can be removed or well controlled; there has no metastasis in other organs; and the patient is generally in good condition and can tolerate surgery. For our patient, there was no chance for surgical excision because of not only the gastric metastasis and adrenal metastasis, but also the enlarged lymph nodes in the abdominal cavity and behind peritoneum. Therefore, surgical excision could not improve her survival.

The median time between breast cancer and initial gastric metastasis is 5 to 7 years.^[24,25]^ Kim et al^[26]^ have reported that gastric metastasis and primary breast cancer are simultaneously detected. Aurello et al^[3]^ have reported a case of gastric metastasis at 14 years after mastectomy for breast cancer. However, the median time from breast cancer to initial adrenal metastasis is 1 to 4 years.^[6,7,9,27]^ In the present patient, the gastric and adrenal metastases occurred at 17 months after the operation of breast cancer. Overall survival of patients with gastric metastasis from breast cancer ranges from 24 to 58 months,^[4,12,25]^ and usually the median overall survival is 2 years since patients with gastric metastasis are prone to metastasis to other sites. The median overall survival of adrenal metastasis is longer compared with gastric metastasis,^[7,9]^ and one-third of these patients, especially for those with surgical resection, can survive for more than 5 years.^[28]^

Our current case report has certain limitation. There was no outcome reported because of the time limitation. We expected to present more prognostic data to help diagnosis and treatment of this rare metastasis from breast cancer.

## 4 Conclusions

Collectively, this was the first reported case of patient with concurrent adrenal and gastric metastases from IDC of the breast. For patients under this condition, we suggested that early recognition and necessary examinations, including gastroscopy and CT, would probably help diagnosis. Of course, pathological and IHC examinations could make accurate diagnosis. Selection of appropriate treatment options could benefit the survival of patients. Apparently, such recommendation was only drawn based on a rare case, and further clinical research is necessary.

## Author contributions

**Project administration:** Tao Zhou.

**Resources:** Lina Zhang, Chunxiao Li.

**Writing – original draft:** Tiantian Tang.

**Writing – review & editing:** Tiantian Tang.

## References

[1] Signorelli C Pomponi-Formiconi D Nelli F Single colon metastasis from breast cancer: a clinical case report. Tumori 2005;91:424–7. 16459641 10.1177/030089160509100509

[2] Jones GE Strauss DC Forshaw MJ Breast cancer metastasis to the stomach may mimic primary gastric cancer: report of two cases and review of literature. World J Surg Oncol 2007;5:1477–85. 10.1186/1477-7819-5-75 PMC1937002 17620117

[3] Aurello P D’Angelo F Cosenza G Gasric metastasis 14 years after mastectomy for breast lobular carcinoma: case report and literature review. Am Surg 2006;72:456–60. 16719204

[4] Almubarak MM Laé M Cacheux W Gastric metastasis of breast cancer: a single centre retrospective study. Dig Liver Dis 2011;43:823–7. 21616731 10.1016/j.dld.2011.04.009

[5] Güler SA Şimşek T Pösteki G A very rare reason for gastric perforation, caused by gastric metastasis of breast cancer: case presentation. Eur J Breast Health 2019;15:59–62. 30816356 10.5152/ejbh.2018.4285 PMC6385715

[6] Fernández Sarabia MT Rodríguez García JM Cardenal Escarcena A Adrenal metastasis of breast cancer with involvement of the inferior vena cava. Clin Transl Oncol 2008;10:761–3. 19015074 10.1007/s12094-008-0284-8

[7] Liu XJ Shen P Wang XF Solitary adrenal metastasis from invasive ductal breast cancer: an uncommon finding. World J Surg Oncol 2010;8:7. 20105336 10.1186/1477-7819-8-7 PMC2824745

[8] Borst MJ Ingold JA Metastatic patterns of invasive lobular versus invasive ductal carcinoma of the breast. Surgery 1993;114:637–41. 8211676

[9] Demirci U Buyukberber S Cakir T Isolated mucinous adrenal metastasis in a breast cancer patient. J Oncol Pharm Pract 2011;17:444–7. 21282299 10.1177/1078155210384893

[10] Piquet F Tibi C Allouch J Gastric metastases of breast cancer. A case of linitis plastica type. Ann Chir 1986;40:333–5. 3035990

[11] Tualllon P Bourgeois M Dargent M Adrenal metastasis in cancer of the breast. Bull Assoc Fr Etud Cancer 1963;50:139–58. 13994649

[12] Koike K Kitahara K Higaki M Clinicopathological features of gastric metastasis from breast cancer in three cases. Breast Cancer 2014;21:629–34. 21779814 10.1007/s12282-011-0284-3

[13] McLemore EC Pockaj BA Reynolds C Breast cancer: presentation and intervention in women with gastrointestinal metastasis and carcinomatosis. Ann Surg Oncol 2005;12:886–94. 16177864 10.1245/ASO.2005.03.030

[14] Li Q Xu BH Li Q Clinical analysis of 34 patients with adrenal metastasis from breast cancer. Zhonghua Zhong Liu Za Zhi 2013;35:855–7. 24447485

[15] Zhang RY Huang SY Li HP Analysis of clinical features and prognosis of breast cancer with uncommon metastases. TUMOR 2017;37:157–62.

[16] Netelenbos T Nooij MA Nortier JW Diabetes insipidus and adrenal insufficiency in a patient with metastatic breast cancer. Neth J Med 2006;64:310–3. 16990696

[17] Wang Z Spaulding B Sienko A Mammaglobin, a valuable diagnostic marker for metastatic breast carcinoma. Int J Clin Exp Pathol 2009;2:384–9. 19158935 PMC2615595

[18] Villarroel Salinas J Ortiz Hidalgo C Soria Céspedes D Immunohistochemical comparison between GCDFP-15and estrogen and progesterone receptors in the diagnosis of metastatic carcinoma of the breast. Gac Med Mex 2012;148:213–7. 22820353

[19] Ni YB Tsang JYS Shao MM GATA-3 is superior to GCDFP-15 and mammaglobin to identify primary and metastatic breast cancer. Breast Cancer Res Treat 2018;169:25–32. 29340880 10.1007/s10549-017-4645-2

[20] Tanvetyanon T Robinson LA Schell MJ Outcomes of adrenalectomy for isolated synchronous versus metachronous adrenal metastases in nonsmall-cell lung cancer: a systematic review and pooled analysis. J Clin Oncol 2008;26:1142–7. 18309950 10.1200/JCO.2007.14.2091

[21] Kanjo T Albertini M Weber S Long-term disease-free survival after adrenalectomy for isolated colorectal metastases. Asian J Surg 2006;29:291–3. 17098665 10.1016/S1015-9584(09)60105-6

[22] Do YR Song HS Kim IH Adrenalectomy for metastatic disease to the adrenal gland from gastric cancer: report of a case. Korean J Intern Med 2007;22:18–20. 17427640 10.3904/kjim.2007.22.1.18 PMC2687596

[23] Yoshitomi S Tsuji H A case of recurrent breast cancer with solitary adrenal metastasis treated with surgery and endocrine therapy. Gan To Kagaku Ryoho 2012;39:2074–6. 23267981

[24] Villa Guzmán JC Espinosa J Cervera R Gastric and colon metastasis from breast cancer: case report, review of the literature, and possible underlying mechanisms. Breast Cancer (Dove Med Press) 2017;9:1–7. 28096693 10.2147/BCTT.S79506 PMC5207330

[25] Hong J Kim Y Cho J Clinical features and prognosis of breast cancer with gastric metastasis. Oncol Lett 2019;17:1833–41. 30675245 10.3892/ol.2018.9754 PMC6341777

[26] Kim DH Son SM Choi YJ Gastric metastasis from invasive lobular breast cancer, mimicking primary gastric cancer: a case report. Medicine (Baltimore) 2018;97:e0258. 29595684 10.1097/MD.0000000000010258 PMC5895432

[27] Andjelić-Dekić N Božović-Spasojević I Milošević S A rare case of isolated adrenal metastasis of invasive ductal breast carcinoma. Srp Arh Celok Lek 2014;142:597–601. 25518541 10.2298/sarh1410597a

[28] Tanvetyenon T Robinson LA Schell MJ Outcomes of adrenalectomy for isolated synchronous versus metachronous adrenal metastases in nonsmallcell lung cancer: a systematic review and pooled analysis. J Clin Oncol 2008;26:1142–7. 18309950 10.1200/JCO.2007.14.2091

